# Determination of Geochemical Bio-Signatures in Mars-Like Basaltic Environments

**DOI:** 10.3389/fmicb.2017.01668

**Published:** 2017-09-08

**Authors:** Karen Olsson-Francis, Victoria K. Pearson, Elisabeth D. Steer, Susanne P. Schwenzer

**Affiliations:** ^1^School of Environment, Earth and Ecosystem Sciences, Open University Milton Keynes, United Kingdom; ^2^School of Physical Sciences, Open University Milton Keynes, United Kingdom; ^3^Nanoscale and Microscale Research Centre, University of Nottingham Nottingham, United Kingdom

**Keywords:** bio-signatures, Mars, life detection, thermochemical modeling, microbial weathering, basalt

## Abstract

Bio-signatures play a central role in determining whether life existed on early Mars. Using a terrestrial basalt as a compositional analog for the martian surface, we applied a combination of experimental microbiology and thermochemical modeling techniques to identify potential geochemical bio-signatures for life on early Mars. Laboratory experiments were used to determine the short-term effects of biota on the dissolution of terrestrial basalt, and the formation of secondary alteration minerals. The chemoorganoheterotrophic bacterium, *Burkholderia* sp. strain B_33, was grown in a minimal growth medium with and without terrestrial basalt as the sole nutrient source. No growth was detected in the absence of the basalt. In the presence of basalt, during exponential growth, the pH decreased rapidly from pH 7.0 to 3.6 and then gradually increased to a steady-state of equilibrium of between 6.8 and 7.1. Microbial growth coincided with an increase in key elements in the growth medium (Si, K, Ca, Mg, and Fe). Experimental results were compared with theoretical thermochemical modeling to predict growth of secondary alteration minerals, which can be used as bio-signatures, over a geological timescale. We thermochemically modeled the dissolution of the basalt (in the absence of biota) in very dilute brine at 25°C, 1 bar; the pH was buffered by the mineral dissolution and precipitation reactions. Preliminary results suggested that at the water to rock ratio of 1 × 10^7^, zeolite, hematite, chlorite, kaolinite, and apatite formed abiotically. The biotic weathering processes were modeled by varying the pH conditions within the model to adjust for biologic influence. The results suggested that, for a basaltic system, the microbially-mediated dissolution of basalt would result in “simpler” secondary alteration, consisting of Fe-hydroxide and kaolinite, under conditions where the abiotic system would also form chlorite. The results from this study demonstrate that, by using laboratory-based experiments and thermochemical modeling, it is possible to identify secondary alteration minerals that could potentially be used to distinguish between abiotic and biotic weathering processes on early Mars. This work will contribute to the interpretation of data from past, present, and future life detection missions to Mars.

## Introduction

The surface of present-day Mars is cold, dry, highly oxidized, and exposed to ultraviolet (UV) and ionizing radiation. These conditions are considered inhospitable; yet early Mars may have had surface conditions more conducive to life, with a possible warmer climate and a denser atmosphere that could provide protection from UV and cosmic radiation (Molina-Cuberos et al., 2001; Bibring et al., 2006; Fairen et al., 2009; Tian et al., 2009). Geological, geochemical, and geomorphological observations made by orbiting spacecraft have provided definitive evidence of more clement and less oxidizing conditions in early martian history (e.g., Carr and Head, 2010; Mangold et al., 2012). Globally and locally, large ancient fluvial systems (including channels and fans) are observed, which may have been habitable (e.g., Malin and Edgett, 2003; Irwin et al., 2005; Mangold et al., 2012; Williams et al., 2013; Fassett and Head, 2015). On Mars' surface, Curiosity has detected complex lake bed stratigraphy, which are reported to contain nitrogen and carbon compounds, including complex organic molecules (Andeer et al., 2013; Williams et al., 2013; Vaniman et al., 2014; Grotzinger et al., 2015; Stern et al., 2015). As a second, independent, strand of evidence for a more hospitable past, the NASA Mars Exploration Rover Opportunity also found evidence for an impact-generated hydrothermal system at Endeavour crater (Arvidson et al., 2014; Fox et al., 2016); impact-generated hydrothermal systems could have provided habitable conditions even during periods of very cold climate (Abramov and Kring, 2005; Schwenzer and Kring, 2009). Mineralogical and geochemical observations of clay minerals, and the detection of carbonates and sulfates, reveal a complex set of environmental conditions ranging from weathering to evaporation, and from cold surface to elevated subsurface temperature conditions, occurring in complex succession (Filiberto and Schwenzer, 2013; Arvidson et al., 2014; Grotzinger et al., 2014, 2015).

On Mars, the surface rocks are dominated by basaltic compositions, but range from ultramafic to basaltic to potentially even more evolved compositions (Nyquist et al., 2001; Christensen et al., 2005; Filiberto, 2008; Treiman and Filiberto, 2015; Morris et al., 2016; Sautter et al., 2016; Mangold et al., 2017). Alteration is known to range from open-system (with and without acidic influence) to closed system conditions, and as a consequence, the resultant rock chemistry may or may not reflect the basaltic source rocks; the products of weathering can be influenced by location, environmental conditions, geological time, and permeability of the rocks at the time of weathering (see Schwenzer and Kring, 2009; Ehlmann et al., 2013; Bridges et al., 2015; Carter et al., 2015; Zolotov and Mironenko, 2016 for example of the wide variety of alteration conditions found on Mars to date). Extensive laboratory based work has demonstrated that microorganisms can use olivine-pyroxene-plagioclase-bearing rocks as a source of bio-essential elements and can enhance the weathering rates (e.g., Berthelin and Belgy, 1979; Vandevivere et al., 1994; Barker et al., 1998; Rogers et al., 1998; Kalinowski et al., 2000; Liermann et al., 2000; Bennett et al., 2001; Welch et al., 2002; Uroz et al., 2009).

The mechanisms employed by microorganisms in basaltic environments are variable and range from the production of excess protons, to the production of low molecular weight organic acids and siderophores (highly specific Fe^(III)^ ligands), and, in some cases, and the production of extracellular polysaccharides and enzymes (Welch and Ullman, 1993; Vandevivere et al., 1994; Barker et al., 1998; Bennett et al., 2001; Wu et al., 2007; Olsson-Francis et al., 2015). These mechanisms may occur in the bulk phase or in local microenvironments, where concentrations would be much higher (Bennett et al., 2001). For example, in terrestrial systems, solutions of organic acids in concentrations comparable to, or slightly higher than, ground water show an increase in dissolution rates of less than one magnitude. In contrast, in local microenvironments the concentration of acid may be much higher (Banfield et al., 1999). If life had once existed on early Mars, it may have left a record of these processes within the martian rock record (Banfield et al., 2001) and recognizing microbially-induced minerals, which could have potentially formed in an early Mars environment, would identify important bio-signatures for use in future life detection efforts.

To ensure unequivocal identification of bio-signatures, comparison with abiotic processes is necessary. Terrestrial abiotic basalt weathering has been extensively studied and characterized using field observations, laboratory-based experiments and thermochemical modeling (e.g., Gislason and Eugster, 1987a,b; Oelkers and Schott, 2001; Wolff-Boenisch et al., 2006). In contrast, to date, stand-alone laboratory experiments have been the only way to investigate the influence that biota has on basalt dissolution and the growth and evolution of secondary alteration minerals (e.g., Wu et al., 2007; Olsson-Francis et al., 2012, 2015). From these experiments alone it is difficult to predict what would happen over years-long or even geological time scales, for example, on early Mars, when the rock is fully dissolved or, more likely, subject to the effects of incongruent dissolution. In these circumstances, the formation of amorphous and leached layers, and secondary mineral precipitation, may occur, influencing the availability of cations for use in biological metabolism.

Themochemical modeling is a powerful tool that aids the prediction of mineral assemblage formation by assessing reaction pathways and studying the formation of secondary minerals in a gas-fluid-rock system (e.g., Reed, 1982; Kühn, 2004) and this has been applied to studies of secondary mineral formation and fluid compositions on Mars (Bridges and Schwenzer, 2012; Filiberto and Schwenzer, 2013; Schwenzer and Kring, 2013; Bridges et al., 2015). In biotic experiments, geochemical models such as Geochemist's workbench are routinely used to characterize mineral precipitation during laboratory-based experiments (e.g., Orcutt et al., 2011; Olsson-Francis et al., 2012), and to characterize redox reactions in water-rock environments (Posth et al., 2008). However, there have been limited, if any, studies that have combined laboratory-based experiments and thermochemical modeling to compare biotically and abiotically generated secondary alteration mineralization over geological time scales.

In this paper, we present preliminary work carried out to investigate the feasibility of coupling microbial dissolution experiments with thermochemical modeling in order to identify mineralogical bio-signatures that could be used as evidence of life on early Mars. The experiments invoke chemoorganoheterotrophic metabolism as a plausible metabolism on early Mars because of the availability of suitable energy sources (electron donors) (Cockell, 2014; Westall et al., 2015). For example, organic carbon at Gale Crater has been predicted to be between 800 and 2,400 ppm. Even if 1% or less of this carbon was bio-available on early Mars this could sustain a chemoorganoheterotrophic community of 10^5^ cells/g of sediment (Sutter et al., 2016). Further, if life had evolved on early Mars, either by photosynthesis or chemolithotrophy, their organic remnants could be used as an electron donor for this metabolism. Finally, chemoorganoheterotrophic metabolism is known to enhance silicate dissolution, predominantly by the production of excess protons and organic acids (Wu et al., 2007; Olsson-Francis et al., 2015).

For microorganisms that only utilize minerals as essential micronutrients and macronutrients (i.e., they are not respired), acidification is the most common, and straightforward, mechanism for silicate dissolution and is generally universal (for review see Uroz et al., 2009). Our understanding of basaltic weathering is predominately based on aerobic metabolism (e.g., Welch and Ullman, 1993; Vandevivere et al., 1994; Blake and Walter, 1996; Drever and Stillings, 1997). On Mars, aerobic metabolism may have been feasible, Curiosity data indicates that there is sufficient molecular oxygen (~1,450 ppm; Mahaffy et al., 2013) in the martian atmosphere to support aerobic activity (King, 2015). In addition, inorganic alteration reactions that are expected to happen due to water-rock interactions have been shown to shift the redox environment to more oxidizing conditions (e.g., Bridges and Schwenzer, 2012). Therefore, traditional experiments in anoxic conditions (e.g., Schirmack et al., 2014, 2015) are complemented by the experiments in this study, conducted in a more oxidizing milieu.

Step by step titrations were modeled to investigate the effect that microbially-mediated acidification has on the aqueous environment and the precipitation of secondary alteration minerals during microbial-mediated basalt weathering. In parallel, abiotic alteration at different pH are investigated to understand the effects of biota by contrasting the two systems. Secondary alteration minerals uniquely produced by microbial-mediated weathering could be used as a geochemical bio-signature for life. The findings of this study are important for further developing geochemical models that could be used to predict bio-signatures for life on early Mars.

## Materials and methods

### Water to rock ratios

For this study, it is critically important to clearly define three different water to rock ratios: we use subscripts E (experiment), D (dissolved), and M (model) to denote the exact meaning of W/R in each context. (W/R)_E_ is the experimental water to rock ratio, simply referring to the ratio of the amount of rock and the amount of water weighed out and mixed for the experiment. This is distinct from (W/R)_D_, which is the amount of rock actually dissolved during the experiment. These two water to rock ratios are different, because, even though dissolution of rock in water is slow, a very small amount of rock will dissolve during the experiment. We assess (W/R)_D_ by looking at the most soluble elements in the fluids resulting from the experiment. (W/R)_M_ is the water to rock ratio used in the model, and assumes complete dissolution of all rock. We compared the (W/R)_D_ and (W/R)_M_ values at the concentration where the most soluble elements are similar.

### Characterization and preparation of mars analog rock

Basalt from Skye was purchased from Richard Tayler minerals (United Kingdom) as an analog for martian basalts. A polished thin section of the rock was prepared for microprobe analysis. The remaining rock was broken with a hammer and pieces devoid of visible weathering were ground using a Tema swing mill, for 8 min. The crushed rock was sieved to select for a fraction size between 250 and 500 μm. Fine particles were removed by ultrasonication in MiliQ water (Olsson-Francis et al., 2010, 2012). The rock was then dried for 24 h, at 80°C. The specific surface area of the ground rock samples was measured using multi-point BET (Brunauer, Emmett and Teller analysis at Imperial College London) with N_2_ and yielded a surface area of 0.976 m^2^ g^−1^.

Petrological analysis was carried out on thin sections of the sample using reflective and transmitted microscopy and a FEI Quanta 3D dual beam scanning electron microscope (SEM) fitted with an Oxford Instruments 80 mm X-Max energy dispersive X-ray spectrometer (EDS), which was operated with an accelerating voltage of 20 kV and a 10–15 mm working distance. The major elemental composition of the basalt was obtained using an ARL 8,420+ dual goniometer wavelength dispersive X-ray Fluorescence (XRF) spectrometer (Applied Research Laboratories, Ecublens, Switzerland). Mineral analysis was obtained from a Cameca SX100 electron microprobe (EMPA) at The Open University using a spot size of 10 μm, accelerated voltage of 20 kV and a beam current of 20 nA.

### Basalt mineralogy

**Figure 1:**
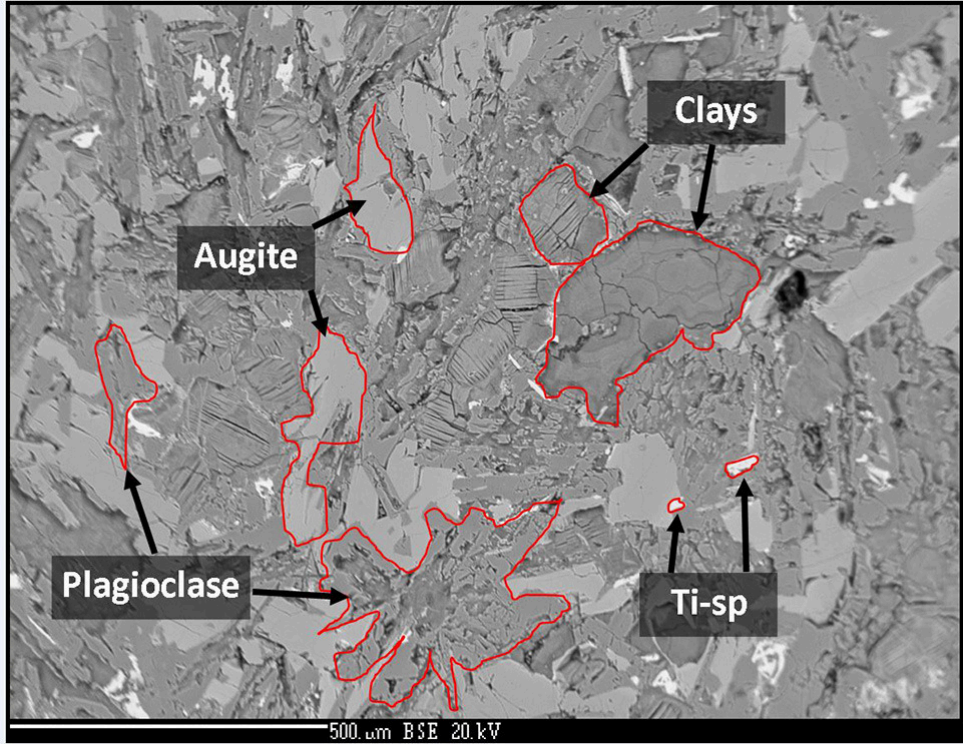
Back-scatter electron image of the analog basalt used in this experiment. Augite, plagioclase, clay, and Ti-spinel are observed. Image width is ~1.5 mm.

The sample was an altered amygdaloidal basalt from the Skye Tertiary Province. The amygdales were filled with zeolites, which were removed from the sample before experimentation commenced. The sample was fine grained with a groundmass of augite and plagioclase with an accessory phase of Ti-spinel. Much of the original mineralogy had been altered at low temperature to form clays and the general mineralogy was characterized as: 20–25% augite, 30% plagioclase, 50% clays. The amount of Ti-spinel was <1%. The plagioclase was present as laths up to 1 mm in size; the augite crystals were largely anhedral and below 0.5 mm in size, as shown in Figure 1. Anhedral grains of Ti-spinel below 100 μm occured throughout the sample. The size and shape of Ti-spinel crystals was prohibitive of EMPA analysis. Representative analyses of the minerals present are detailed in Table 1.

**Table 1:** Representative analyses of minerals in the basalt (wt %).

	**SiO_2_**	**Al_2_O_3_**	**TiO_2_**	**MgO**	**CaO**	**MnO**	**FeO**	**Na_2_O**	**K_2_O**	**Total**
Augite	50.63	3.25	1.11	14.51	20.59	0.20	8.83	0.33	0.00	99.45
Plagioclase	58.27	25.63	0.11	0.10	8.45	0.00	0.74	6.68	0.52	100.50
Zeolite	50.69	23.49	0.00	0.01	10.63	0.01	0.02	1.63	0.47	86.95
Vermiculite	34.97	11.84	0.00	13.04	1.45	0.32	20.78	0.09	0.04	82.53
Saponite	41.10	11.54	0.00	17.65	0.71	0.25	13.13	0.09	0.04	84.51

**Figure 2:**
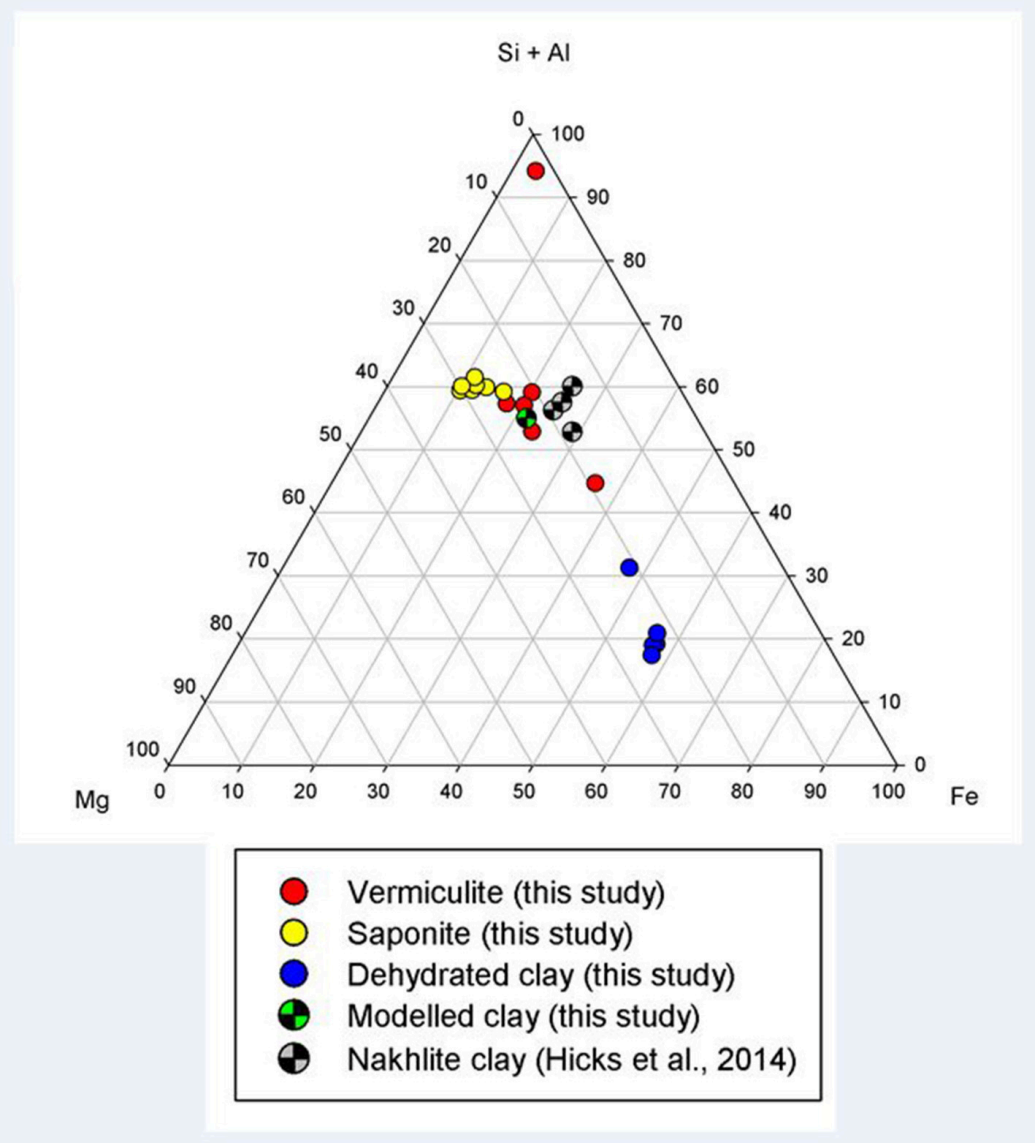
Ternary plot of (Si+Al)–Mg–Fe showing the clay mineral compositions observed in this study (filled circles), in the models of this study (checkered green circle), and in the nakhlite martian meteorites (checkered gray circle; Hicks et al., 2014).

The clays present in the sample were vermiculite and saponite. These are similar in composition to clays found by the Curiosity rover on Mars by XRD (Vaniman et al., 2014) and to those modeled by Bridges et al. (2015) to be present in Gale Crater. As a comparison, analyses from this study have been plotted with clay compositions modeled to exist on Mars by Bridges et al. (2015) and with clays measured in the martian Lafayette meteorite by Hicks et al. (2014). Figure 2 shows that the vermiculites and saponite identified in this study form a tight cluster that overlaps with the martian clays as well as the clays modeled here. However, the range of terrestrial clays extends beyond the narrow range of isochemical alteration, showing more Si and Al rich endmembers and clays depleted in those two elements. The latter could be due to an oxidizing reaction causing microcrystalline Fe-oxides and hydroxides intermixed with the clay. The mixture of clay and pristine mineral remnants makes this basalt a proxy for the recently discovered “mudstones” (Vaniman et al., 2014), of basaltic origin, on Mars.

### Model bacterium

The microbiological experiments were conducted using the bacterium *Burkholderia* sp. strain B_33, which has previously been isolated from nutrient limiting soil, in Snowdonia National Park, United Kingdom (Olsson-Francis et al., 2015). A *Burkholderia* sp. was selected because long-term experiments in nutrient poor soils have shown a correlation between the abundance of this genus and specific minerals, such as plagioclase and a mix of phlogopite and quartz (Uroz et al., 2009; Lepleux et al., 2012). It also exhibits chemoorganoheterotrophy, a plausible metabolism for life on past Mars.

Routine growth was carried out in a modified minimal medium, which contained the following (mg L^−1^): 10 of FeCl_3_, 150 of MgSO_4_•6H_2_O, 20 of CaCl_2_; 20 of KCl, 65 of NH_4_Cl, 100 of NaNO_3_, 70 of K_2_HPO_4_, 60 of KH_2_PO_4_, 20 of glucose. All of the reagents, except the MgSO_4_•6H_2_O and the FeCl_3_ were added and the pH was adjusted to pH 7.0. After the medium was autoclaved (20 min at 121°C), filtered sterilized MgSO_4_•6H_2_O and FeCl_3_ were added (this did not alter the pH).

### Basalt dissolution experiment

The dissolution experiments were carried out in batch culture. The growth medium contained the following: 2 g L^−1^ of glucose, 0.06 g L^−1^ of NH_4_Cl, and 200 g L^−1^ of basalt. Twenty grams of the crushed basalt (0.5–1 mm) was placed in an acid-washed 125 ml glass Erlenmeyer culture flask and autoclaved at 121°C for 15 min. Two g L^−1^ of glucose was added ensure that growth was not limited by the amount of carbon. One hundred milliliters of autoclaved liquid medium was added to the flask and the pH was adjusted to pH 7.0 with filtered sterilized 10 mM NaOH. The experiments were conducted with a water to rock (W/R)_E_ ratio of 100/20 (BET surface area of 0.976 m^2^ g^−1^).

Prior to inoculating the flasks, cells were washed to remove any excess growth medium. Ten milliliters of exponentially grown cells were harvested by centrifugation at 4,000 × g, for 5 min. The cell pellet was washed three times with sterilized 50 mM Tris buffer (pH 7.0) and re-suspended to a final cell density of 10^7^–10^8^ cell mL^−1^. A 0.5% inoculum was used to inoculate the flasks. The flasks were incubated, without shaking, at 25°C for 28 days. Each of the experiments were carried out in triplicate. The biotic experiments were designated B1, B2, and B3, and the abiotic controls were designated C1, C2, and C3. Abiotic controls were prepared in an identical manner to the biotic flasks.

At day 1, 4, 7, 11, 14, 21, and 28, 5.5 mL aliquots were aseptically removed from the flask and transferred to a 10 mL acid-washed bottle. Each aliquot was immediately processed as follows: 4.5 mL was passed through a 0.2 μm nylon syringe filter and acidified with concentrated HNO_3_ (final 5% acid) for elemental concentration measurements and 1 mL was left unfiltered and used to measure microbial growth and pH.

### Measuring bacterial growth

Cells were stained with the nucleic acid-binding dye SYBR Green I DNA (0.1% w/v stock; Life Technologies, Paisley, UK) and 1 mL of culture was filtered through a 0.2 μm black polycarbonate filter and then washed with 100 μL of sterile dd H_2_O. The cells on the filter were enumerated using a Leica DMRP microscope equipped with epifluorescence, as previously described (Summers et al., 2013). The growth rate constant (*k*) for the log phase of growth was determined (Pirt, 1978).

### Siderophore production

To determine the ability of the isolates to produce siderophores, the Chrome Azurol S liquid assay (CAS) was used (Schwyn and Neilands, 1987). As a control we used *Cupriavidus metallidurans* CH34, which has previously been shown to produce siderophores under iron limiting conditions (Olsson-Francis et al., 2010). The detection of siderophores was quantified and defined, as previously described (Payne, 1994). The isolates were grown in the minimal medium, without iron (i.e., they were iron-limited), and siderophore production was measured in stationary stage cells.

### Chemical analyses

The pH was measured using an Orion 3-Star Thermo Scientific bench top meter with an uncertainty of 0.01 pH unit. The total elemental concentration of dissolved elements in the growth medium was measured using Inductively Coupled Plasma–Mass Spectrometry (ICP-MS; Agilent 7500s ICP-MS with New Wave 213 laser system). Detection limits for the ICP-MS were as follows: Ca (39.89 nmol L^−1^), Mg (2.81 nmol L^−1^), P (236.64 nmol L^−1^), Fe (41.83 nmol L^−1^), Na (7.59 nmol L^−1^), Si (236.63 nmol L^−1^), and Al (2.03 nmol L^−1^). Each of the measurements were conducted in triplicate and the mean was reported; the standard deviation was <5%. The data was corrected for the decrease in fluid volume and the loss of elemental mass during sampling, as previously described (Wu et al., 2007). Glucose concentration was measured using the Amplex red glucose kit (Invitrogen). The absorbance was measured at 595 nm and compared with a calibration curve of known glucose concentrations. Oxalate was measured using an oxalate oxidase assay (Trinity Biotech), at 590 nm, as per manufacturer's instructions.

### FEG-SEM analysis

After 28 d, rocks were removed from the flasks for Field Emission Gun (FEG)-SEM analysis. The rocks were air dried and carbon coated (15–20 nm thickness) on aluminum stubs. The surface of the rocks was examined using a using a FEG-SEM with an EDS detector (ZEISS Supra; 55-VP; Zeiss Microimaging, Göttingen, Germany), which was operated with an accelerating voltage of 2–15 kV and a 7–10 mm working distance.

### Elemental release rates

The kinetics of elemental release were calculated using the linear release rate ($R_{i}^{l}$), as previously described (Wu et al., 2007). Any significant difference between the biotic and abiotic dissolution kinetics were identified using a Student's *t*-test.

### Elemental uptake

The intracellular elemental concentration was measured at day 28. Twenty milliliters of liquid culture was aseptically taken from the biotic experiments and centrifuged at 13,000 × *g* for 20 min. The resulting cell pellet was washed three times in sterilized 0.5% HNO_3_. The cells were washed to ensure that no elements from the experimental solution were analyzed; however, we cannot rule out that elements that were loosely bound to the cells were not lost during this step. The pellet was dried at 80°C overnight, and digested in 1 mL concentrated HNO_3_ (final 5% acid) and the resulting solution was analyzed by ICP-MS, as previously described (Olsson-Francis et al., 2012).

### Thermochemical modeling

In order to assess the inorganic reaction pathways possible in the rock-fluid system, we used thermochemical modeling, specifically the code CHIM-XPT (previously CHILLER Reed and Spycher, 2006; Reed et al., 2010). This code has been used extensively in terrestrial basaltic environments (e.g., Reed, 1982) and applied to basaltic rocks of martian compositions (e.g., Debraal et al., 1993; Schwenzer and Kring, 2009; Filiberto and Schwenzer, 2013; Bridges et al., 2015; Schwenzer et al., 2016). We carried out stepwise titration simulations from the initial fluid to a (W/R)_M_ of 1 to assess dissolution of the rock in the fluid without biotic activity, starting at a very high (W/R)_M_ of over 10^8^, modeling titration to a low (W/R)_M_ of 1. We report data from (W/R)_M_ of 1 Mio ensuring that constant conditions independent of any starting mineral choices had been reached at this point. Input data included the rock composition as derived by XRF, and the fluid composition of the minimal growth medium (Table 2). Note that the fluid composition is summarized in Table 2 as one species per element, but elements will be partitioned between several species as relevant to the pH and Eh conditions in the fluid during the modeling. For example, Fe is partitioned between Fe^2+^ and Fe^3+^ species according to redox conditions in the respective modeling step, and compound species, e.g., FeOH or FeCl-bearing ions, are also considered important in this respect. Overall, a set of ~80 different ionic species are typically used to represent the fluid chemistry in each calculation iteration within the modeling steps. We model at 1 bar and 25°C, which closely mimics the pressure and temperature conditions of the growth experiment and investigate three model experiments: In the first experiment, pH varies, and is treated as a free parameter. In two subsequent experiments, pH is set to 7 and 4, respectively, to simulate the conditions during the stationary and exponential phases in the growth experiment. The Cl-anion is used for charge balance, which is especially important for the pH 4 model, because additional Cl^−^ is required to set the initial pH.

**Table 2:** Input data for the model.

**Rock composition [wt. %]**		**Fluid composition [10^−7^ moles]**	
SiO_2_	51.81	Cl^−^	51.7
Al_2_O_3_	14.21	SiO_2 (aq)_	0.34
Fe_2_O_3_	13.09	Al^3+^	0.37
MgO	4.79	Ca^2+^	0.21
CaO	4.97	Mg^2+^	0.38
Na_2_O	3.83	Fe^2+^	0.01
K_2_O	0.19	K^+^	0.24
MnO	0.22	Na^+^	49.8
P_2_O_5_	0.38	${\text{HPO}}_{4}^{2-}$	0.32

*Species in the fluid are summarized in the table as one species, but during the modeling were partitioned into several dissolved species (for details see text)*.

In the model, we assumed complete dissolution of the rock into solution, which is a simplification many codes make (for an explanation and overview of the diversity of thermochemical rate modeling see Kühn, 2004; Ganguly, 2008; Rimstidt, 2014). To account for a more complex natural system, e.g., varying dissolution rates of the minerals in the rock and/or incongruent dissolution of minerals, the input parameters were varied systematically and with close reference to available thermodynamic data, and experimental and natural observations and data. This results in a parameter space that can be reduced to the most likely approximation of the natural system.

## Results

### Bacterial growth

**Figure 3:**
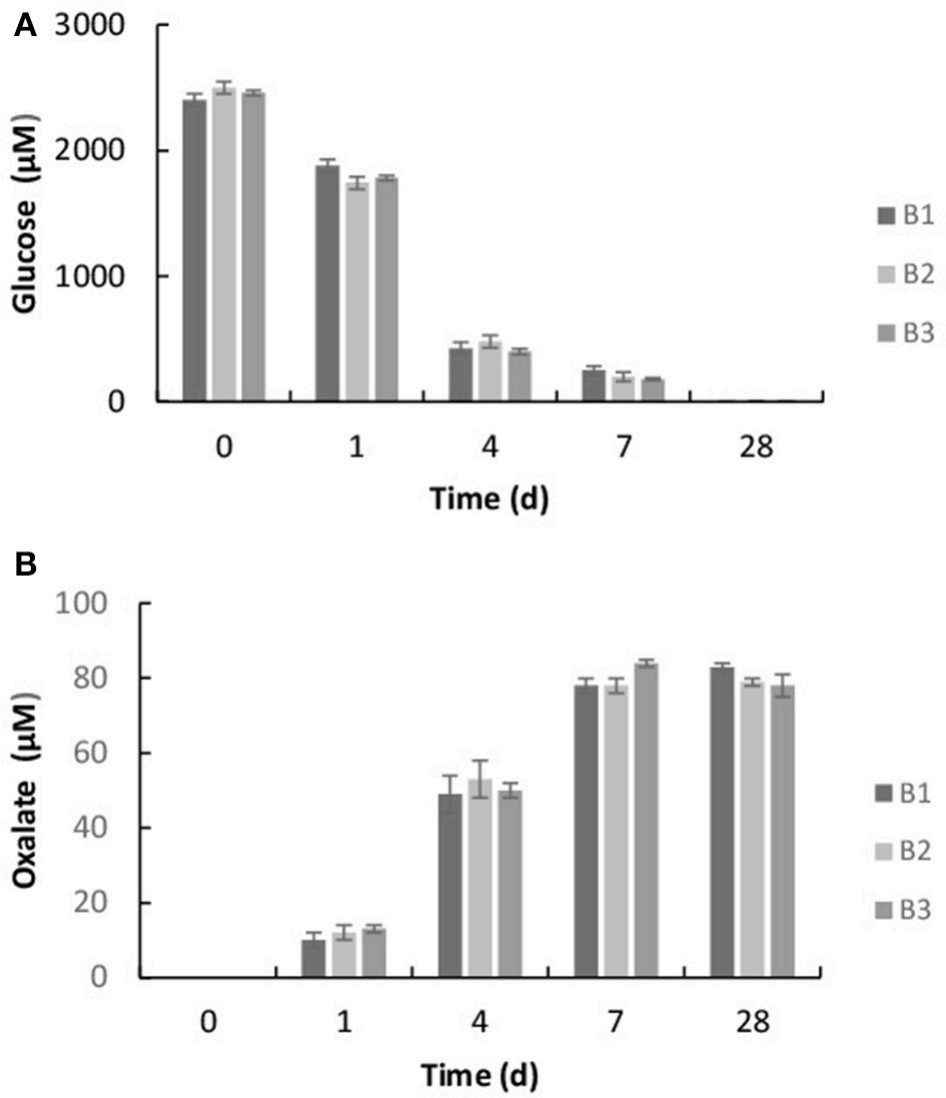
Concentration of glucose **(A)** and oxalic acid **(B)** in the growth medium after 1, 4, 7, and 28 days. The values reported are the means of three independent experiments, and the standard error associated with these determinations is shown.

The bacterium, *Burkholderia* sp. strain B_33 was able to grow in the minimal medium with basalt as the sole source of bio-essential elements. No growth was detected in the growth medium without basalt. The bacterium utilized the glucose within the growth medium as a source of carbon and within 7 days the concentration of glucose decreased to ~20%, as shown in Figure 3A. As the bacterium utilized the glucose, the metabolite oxalate was produced. The concentration of oxalate in the growth medium increased steadily until day 7 and then plateaued (Figure 3B). There was no significant difference between the triplicate flasks.

**Figure 4:**
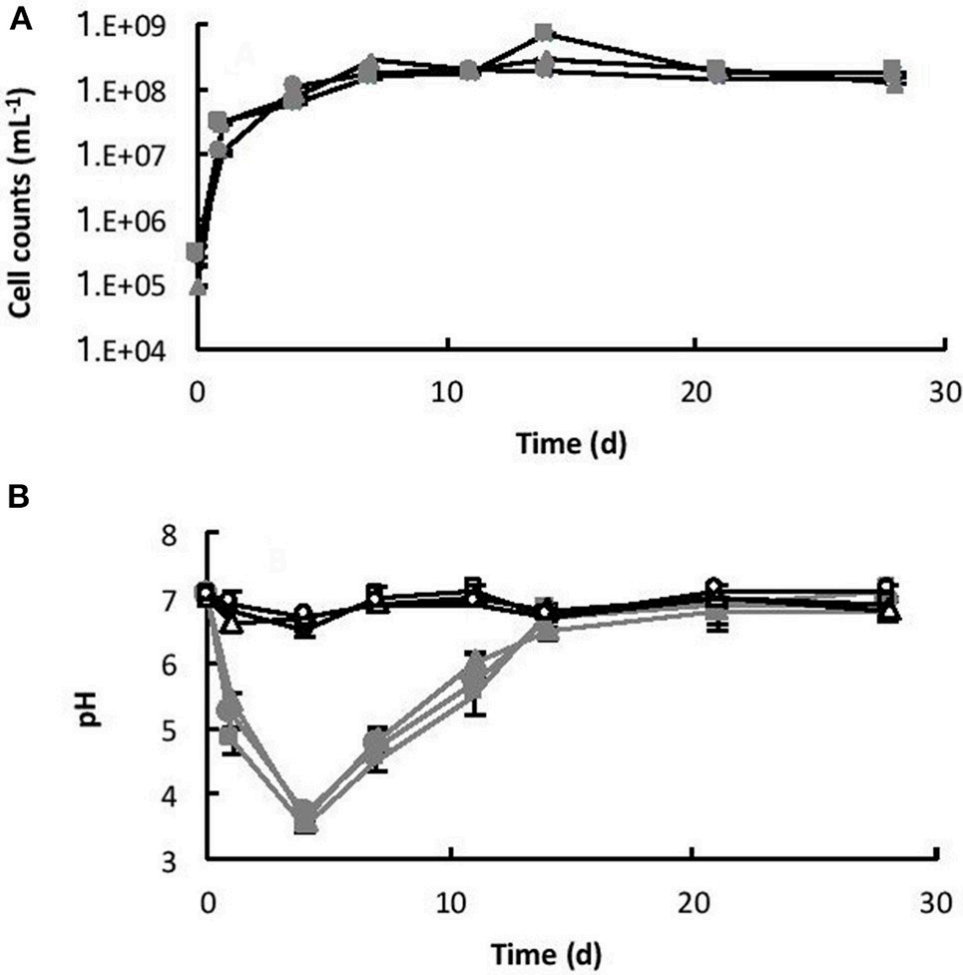
**(A)** Growth of *Burkholderia* sp. strain B_33 in a minimal medium with basalt as the sole source of bio-essential elements. **(B)** Change in pH of the medium over time. The values reported are the means of three independent biotic [B1 (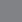), B2 (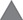), B3 (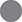)] and abiotic [C1 (□), C2 (△), C3 (○)] experiments, and the standard error associated with these determinations is shown. Cell counts demonstrated that there was no bacterial contamination in the abiotic controls after 28 days.

The initial cell counts, immediately after inoculation, were between 9.23 × 10^4^ (B3) and 2.92 × 10^5^ (B2) cell mL^−1^. Within 7 days, the number of cells had increased to a maximum of between 1.53 × 10^8^ (B2) and 2.82 × 10^8^ (B3) cell mL^−1^ (exponential growth) and then remained relatively steady until the end of the experiment (stationary phase), as shown in Figure 4A. Coinciding with exponential growth, the pH decreased rapidly and at day 4 the pH was between 3.5 (B2) and 3.6 (B3) (Figure 4B). As the cells reached late exponential stage the pH values gradually increased to a steady-state equilibrium of between pH 6.8 and 7.1. In contrast, the pH in the abiotic controls remained near neutral pH (between pH 6.6 and 7.1) throughout the experiment (Figure 4B). No cells were observed in the abiotic controls.

### Siderophore production

*Burkholderia* sp. strain B_33 was screened for siderophore production using the Chrome Azurol S assay. In the minimal medium, without iron, siderophores were produced. The amount varied between 1.20 and 2.10 μmol L^−1^ EDTA equivalent. However, when basalt was added to the growth medium, siderophores were not detected (data not shown).

### Basalt dissolution

**Figure 5:**
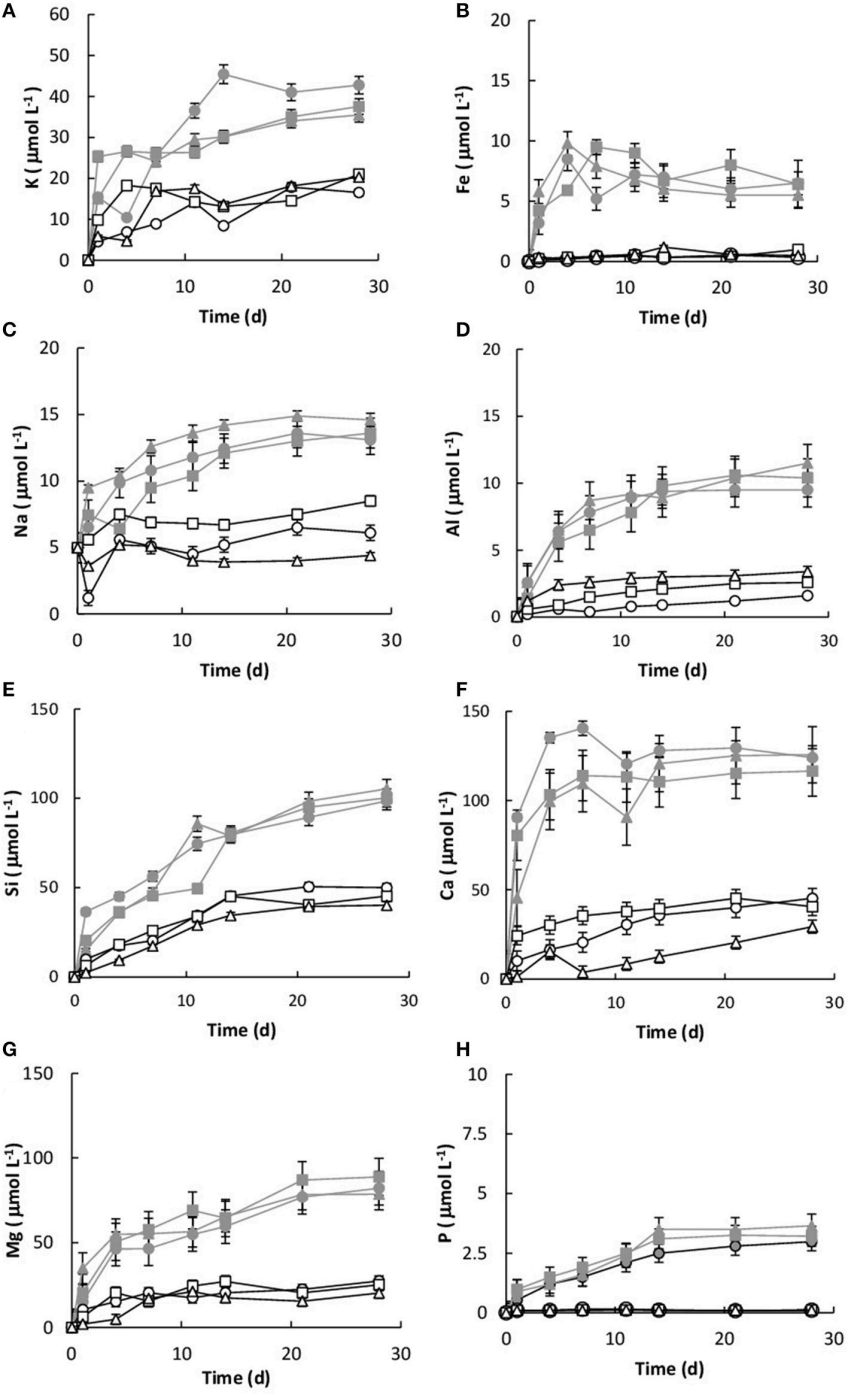
Concentration of K **(A)**, Fe **(B)**, Na **(C)**, Al **(D)**, Si **(E)**, Ca **(F)**, Mg **(G)**, and P **(H)** over time for both the biotic [B1 (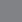), B2 (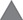), B3 (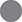)] and abiotic flasks [C1 (□), C2 (∆), C3 (○)]. The values reported are the means of three independent experiments, and the standard error associated with these determinations is shown.

Dissolution was measured by the concentration of key elements (Si, K, Ca, P, Mg, and Fe) within the growth medium. All of the values were corrected for the decrease in fluid volume and the loss of elemental mass during sampling. Without the basalt, the concentrations of Si, K, Ca, Mg, Na, Fe, and P in the growth medium were 0.034, 0.024, 0.021, 0.038, 0.018, and 0.01 μmol L^−1^, respectively. Figure 5 shows the elemental concentrations in the dissolution experiments as a function of time. The presence of *Burkholderia* sp. strain B_33 enhanced the release of Si, Ca, Mg, Al, K, Na, P, and Fe into the growth medium. In general, the concentration increased linearly until day 4–11 (depending on the element) and then they either reached or began to approach steady-state conditions.

**Figure 6:**
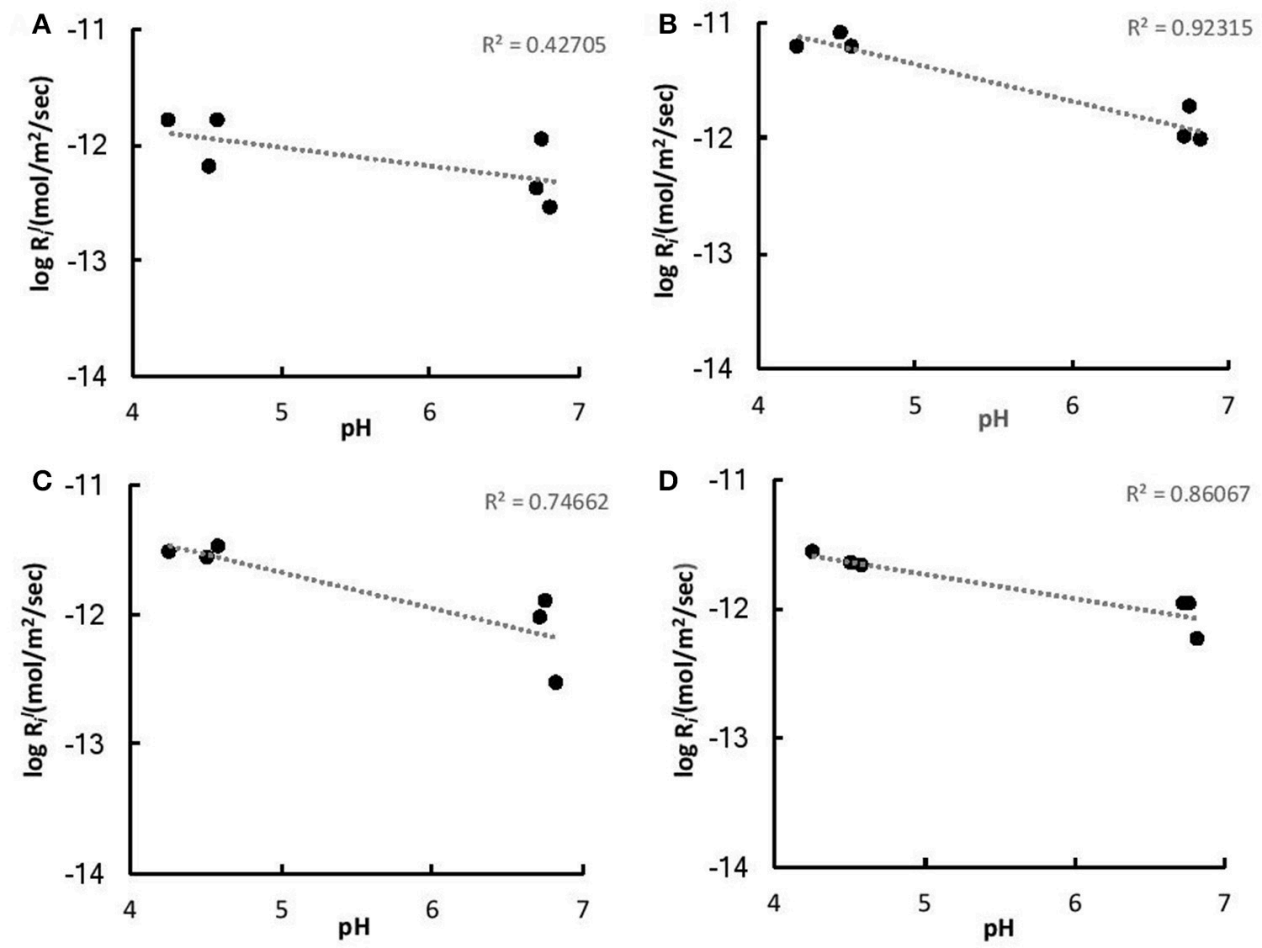
Log$R_{i}^{l}$ values of K **(A)**, Ca **(B)**, Mg **(C)**, and Fe **(D)** vs. the average pH of the initial growth phase (days 1–7).

The initial linear release rates were calculated to compare the dissolution kinetics in the abiotic and biotic flasks, as demonstrated in Table 3. The presence of *Burkholderia* sp. strain B_33 had a significant (*p* < 0.05) effect on the release rates of Si, Ca, Mg, Al, and Fe (there was no significant effect on the release of P, Na, and K). For example, for B1, the $R_{i}^{l}$ value for Ca was approximately eight-fold higher than the abiotic controls. Plotting the log$R_{i}^{l}$ values for Mg, Ca, Si, and K against the average pH of the initial growth phase (day 1–7, which corresponds to the period used to determine the $R_{i}^{l}$ values) suggests that the release rates were dependent on pH (Figure 6).

**Table 3:** Linear elemental release rates $R_{i}^{l}$ calculated from the linear rate law (Wu et al., 2007).

$R_{i}^{l}$ **(10**^**−12**^ **mol m**^**−2**^ **s**^**−1**^**)**								
	**P**	**Si**	**Al**	**Mg**	**Ca**	**Na**	**K**	**Fe**
**BIOTIC**								
B1	0.52	2.68	0.38	2.75	8.02	0.28	0.62	0.51
B2	0.45	2.16	0.21	2.98	6.12	0.08	1.57	0.39
B3	0.42	2.13	0.38	3.26	5.90	0.32	1.56	0.58
**ABIOTIC**								
C1	0.35	1.04	0.03	0.91	0.98	0.03	0.41	0.01
C2	0.32	1.08	0.05	1.22	1.79	0.01	1.08	0.02
C3	0.41	0.56	0.01	0.29	0.92	0.01	0.28	0.01

### Cellular element uptake

The intracellular elemental concentration was measured in bacteria collected from day 28. The values in Table 4 are reported as the elemental concentration per cell. This data was used to determine if elemental uptake was significant with respect to the solute concentrations at day 28. The values reported in Table 3 were multiplied by the number of cells measured at day 28 and the values were corrected for the decrease in fluid volume during sampling, as previously described (Wu et al., 2007; Olsson-Francis et al., 2012). The intracellular elemental concentrations were <1% of the final fluid concentrations.

**Table 4:** Chemical composition of bacterial cells after 28 days.

**Chemical composition [10^−10^ μmol/cell]**							
	**Si**	**Al**	**Mg**	**Ca**	**Na**	**K**	**Fe**
B1	0.02	B.D	0.12	0.01	0.01	0.09	B.D
B2	0.04	B.D	0.09	0.02	0.02	0.08	0.03
B3	B.D	B.D	0.13	0.05	B.D	0.10	0.01

*B.D is below the detection limits*.

### Secondary alteration minerals

**Figure 7:**
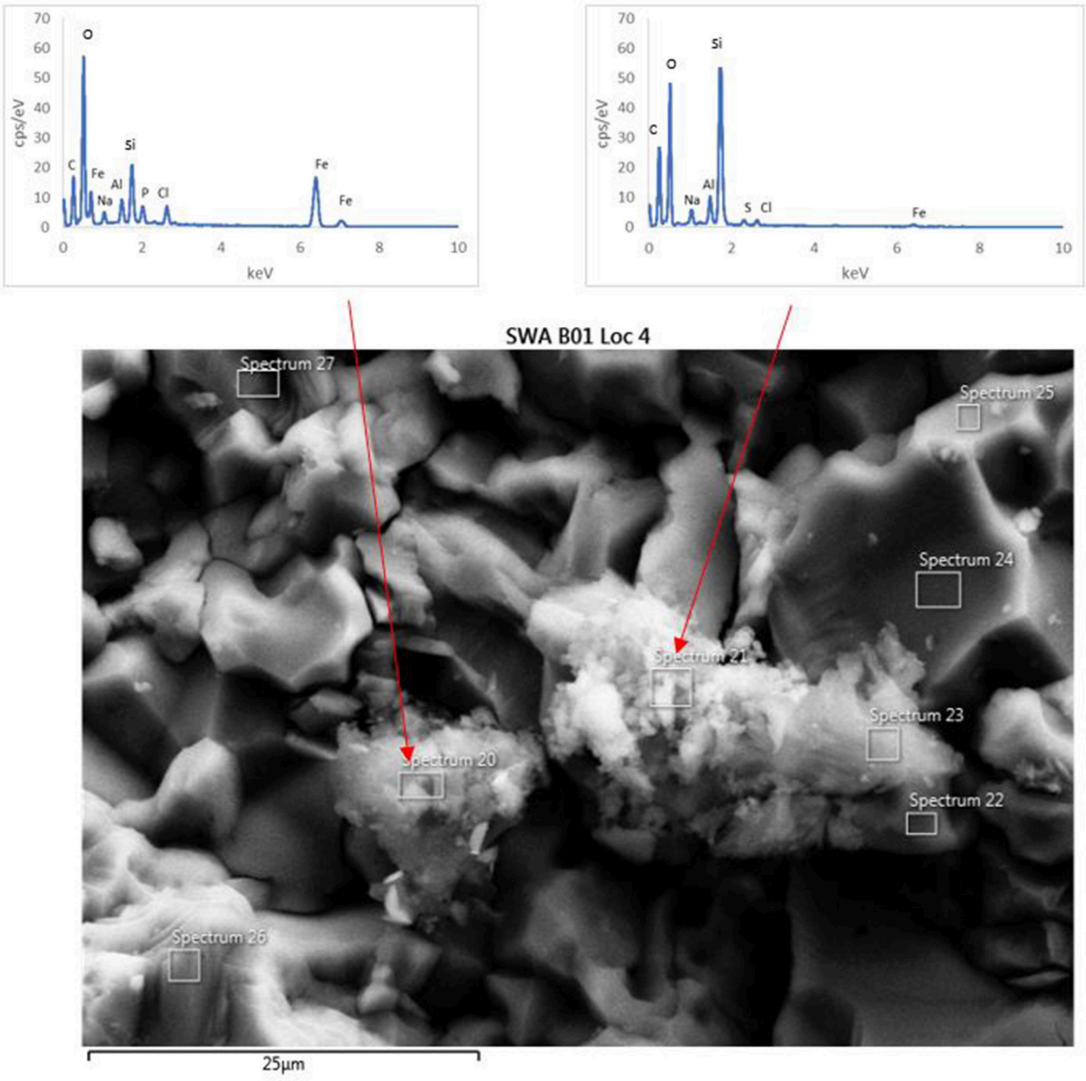
FEG-SEM micrograph of a typical secondary minerals produced in the biological flasks after 28 days. No secondary minerals were observed in the abiotic controls.

The surface of the basaltic rock was examined for secondary alteration products with FEG-SEM and EDS analysis. Where possible, EDS analysis was performed on areas of the rock where secondary alteration was visible on the surface (Figure 7) i.e., where there was a positive relief and evident morphological differences to the crystalline basalt and the clay minerals already contained within the basalt. However, there was limited evidence of alteration on any mineral surfaces; mineral grains from the biotic and abiotic flasks showed predominantly pristine surfaces with the exception of dissolution pits and an amorphous silicate layer on the surface of the rocks in the biotic flasks. Figure 7 also shows small pockets of alteration products in the biotic flasks, which are remarkably diverse (Table 5).

**Table 5:** EDS analysis of two alteration mineral assemblages (refer to spectra 20 and 21 as shown in Figure 7).

**Wt. % element measured^a^^,^^b^^,^^c^**			**Wt. % oxide^a^^,^^b^^,^^c^**			**Wt. % species partitioned^a^^,^^b^^,^^c^**		
	**20**	**21**		**20**	**21**		**20**	
O	43.6	30.3						
Na	3.5	2.3	Na_2_O	4.7	3.1	Na_2_O	2.9	N.D
Al	5.6	3.8	Al_2_O_3_	10.6	7.2	Al_2_O_3_	10.6	7.2
Si	40.1	10.1	SiO_2_	85.8	21.6	SiO_2_	85.8	21.6
Fe	2.9	45.1	FeO	3.7	58.0	FeO	N.D	57.1
Ti	0.6	N.D	TiO_2_	1.0	N.D	TiO_2_	1.0	N.D
P	N.D	3.5	P_2_O_5_	N.D	8.0	P_2_O_5_	N.D	8.0
S	1.7	0.4	SO_2_	3.4	0.8	SO_2_	0.1	N.D
Cl	2.1	4.4	Cl	2.1	4.4	NaCl	3.5	5.8
						FeS	4.6	1.1
						Cl	N.D	0.9
						Al_2_Si_2_O_5_(OH)_4_	N.D	N.D
						Na_2_O	N.D	N.D
Sum	100.1	99.9	Sum	111.3	103.1	Sum	108.3	101.7

a *All Cl is calculated as NaCl, all Fe as FeS, and the remainder of the S as SO2*.

b *All Na is calculated as NaCl with the remaining Cl as element. This analysis has significant P, which could form apatite and host Cl*.

c *In addition to the partitioning in a, Al is calculated as kaolinite. N.D is not detected*.

*Data are given as measured weight % of element converted to oxides, or partitioned into more likely phases, for example, NaCl (for details see text). Zero refers to partitioning results, where an element is calculated as a different species and therefore zero by calculation*.

The mineral assemblage labeled “spectrum 20” is dominated by Si, with minor Al, Na, Fe, S, and Cl. Dividing this analysis into element-oxides results in a “high total,” >100%. Even if it is assumed that Cl is more likely to be bound in CaCl, and all Fe is FeS, the total remains above 100%. Inspection of Figure 7 and knowledge from our models (see below) allows us to assume that kaolinite [Al_2_Si_2_O_5_(OH)_4_] might be present, and if this accounts for all of the Al, the “high total” is reduced further (Table 5). Therefore, it is likely that the alteration assemblage on “spectrum 20” contains silica and kaolinite with minor FeS and NaCl. Alternatively, instead of NaCl, some minor amount of carbonate could take up the excess cations, but is considered unlikely in this partitioning exercise because of the Si-dominance of the precipitate and the amounts of Cl in solution.

The adjacent alteration mineral assemblage has a different composition, since it is even more dominated by Si and Fe, has around 4% Cl, P, and Al, and minor Na and S. The total, after partitioning into oxides, is only slightly above 100%, but is within 2% error of 100% if all Na is assumed to be NaCl and all S is FeS. According to our models, the remaining Fe could be in nontronite [(CaO_0.5_,Na)_0.3_${\text{Fe}}_{2}^{3+}$(Si,Al)_4_O_10_(OH)_2_·nH_2_O]. This analysis also has P (Table 5), which could explain the high Cl; there is significantly Cl more than would be taken by NaCl suggesting the presence of another phase that could take both of these elements. We considered Cl-apatite, which also occurs in our models of this system. Unfortunately, Ca was not evident in our analyses. But with the very small grain size used in the experiments, precision of such results is limited. We therefore note the similarity of some of those results with the models, but do not wish to over-interpret them (which is why we have not balanced the amount of kaolinite to result in a 100% analysis total for point 20 in Table 5).

Any differences between the mineralogy before and after the experiment were below the detection limits for such changes with XRD, which is readily explained by the very small amounts of dissolution and volume of alteration phases observed.

### Thermochemical models—three cases

A set of thermochemical models was used to simulate rock dissolution without bacteria (with pH as free parameter), and to better understand the reactions at the pH conditions invoked by the presence of bacteria during the growth phase (pH 4 and 7; see Section Materials and Methods). We have shown that our model runs within a range of 1,000 > (W/R)_M_ > 2 Mio, but focus our results and discussion on the range between 100,000 and 1 Mio. This water to rock ratio range is considered to be representative of all (W/R)_D_ observed in the biotic experiments. Note, that the overall amounts of precipitated minerals in all modeled scenarios are very small and are typically in the order of 0.1–0.3 mg L^−1^ solution at (W/R)_M_ of 1 Mio and reach ~1 g at (W/R)_M_ of 1,000. Variation in the amount of precipitated phases results from the varying solubility of species under different conditions, but also from structurally bound water in the precipitating phases. Further, we use “quartz” for the precipitation of any of the SiO_2_ polymorphs: quartz, amorphous silica, and chalcedony.

### Abiotic modeling—pH as free parameter

**Figure 8:**
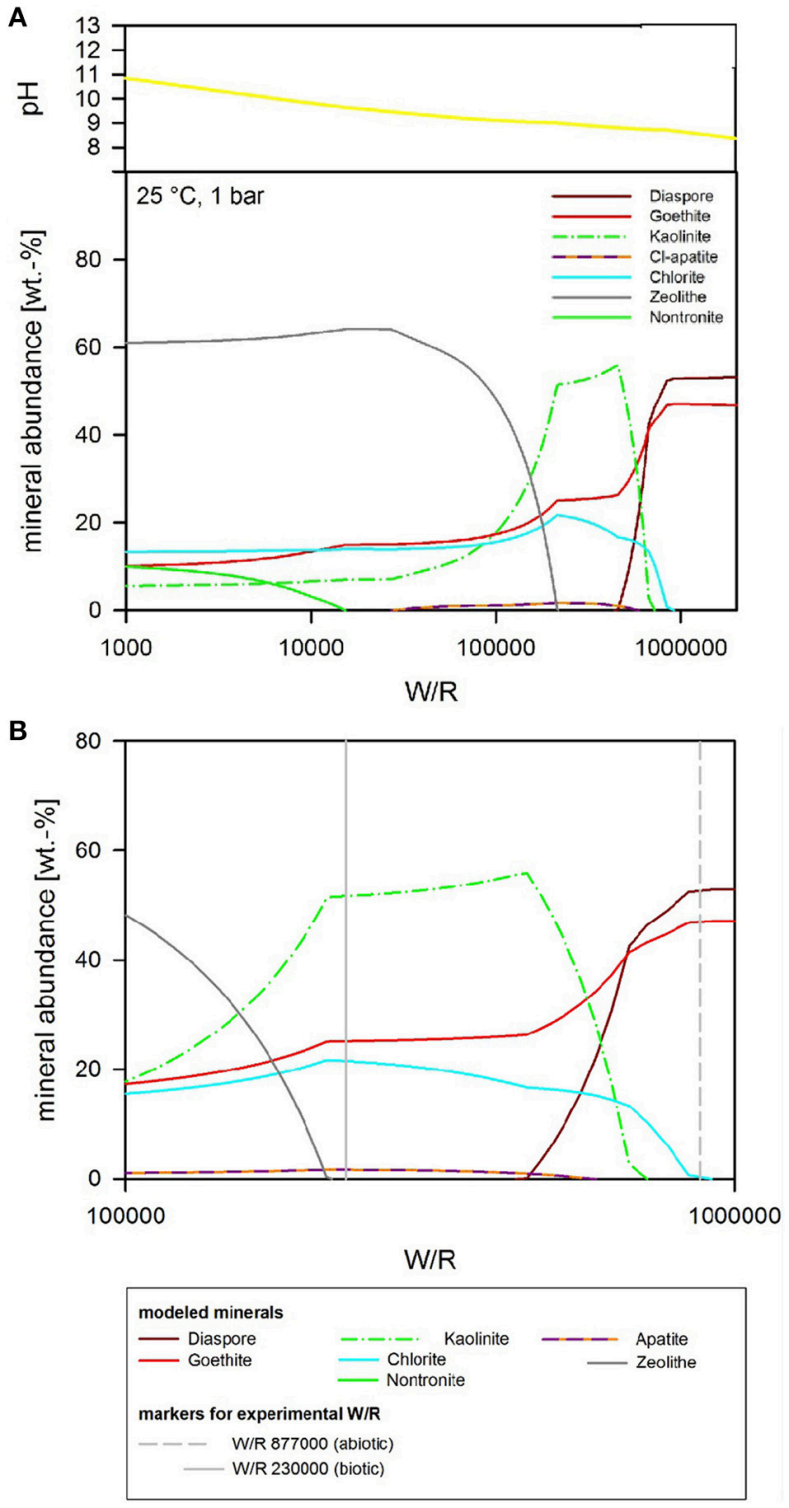
**(A)** Secondary minerals predicted from the dissolution of analog basalt at 25°C with pH as a free parameter. The diagram shows the formation of diaspore, goethite, kaolinite, Cl-apatite, chlorite, zeolite, and nontronite. **(B)** Secondary minerals predicted from the dissolution of analog basalt at 25°C with pH as a free parameter [at a higher resolution than **(A)**]. The (W/R)_D_, represented by the vertical lines, was calculated based on the element potassium because it is not incorporated into any of the expected mineral precipitates and the intracellular concentration was <2%.

If pH is not held constant in the modeling run, it is calculated together with the other parameters from the dissolution and precipitation processes in the system. The fluid invoked has a neutral pH, which is maintained in the very initial dissolution steps but over the course of the titration changes to alkaline (Figure 8). At (W/R)_M_ of 1 Mio, a mixture of diaspore [α-AlO(OH)] and goethite [FeO(OH)] precipitate. With increasing rock dissolution, Al is no longer precipitated as hydroxide but instead consumed by sheet silicate formation. Chlorite [(Mg,Fe^2+^,Mn)_5_Al(AlSi_3_O_10_)(OH)_8_] and kaolinite form alongside goethite, while diaspore is no longer part of the precipitate. Phosphorus is precipitated as chlorapatite [Ca_5_(PO_4_)_3_Cl] as soon as the concentrations of Ca and P are sufficiently high in the fluid for to reach the saturation of apatite. With further dissolution, zeolite [mainly stilbite, NaCa_4_(Si_27_Al_9_)O_72_·28(H_2_O)] formation sets in, resulting in a zeolite–hematite–chlorite–kaolinite–apatite assemblage at (W/R)_M_ of 100,000. With further dissolution, the smectite-group silicate non-tronite is added to the assemblage. As shown above, some of those minerals have been observed in the biotic alteration assemblages, which supports the model results. To observe the full alteration assemblage or fully quantify it using mineralogical methods rather than chemistry, a longer experimental run with more progressive alteration mineral formation would be needed, because quantities are very low. In theory, ~0.05 g of kaolinite would be expected to form at a W/R of 1,000 per 1 g of dissolved basalt in the model, translating to very small amounts of material expected in the experiment. Given the total amount of basalt in the experiment (20 g) and the very limited dissolution observed, we expect alteration phases to be in the ng range, distributed over 20 g of basaltic grains.

### Biotic simulation—pH buffered at pH 7

**Figure 9:**
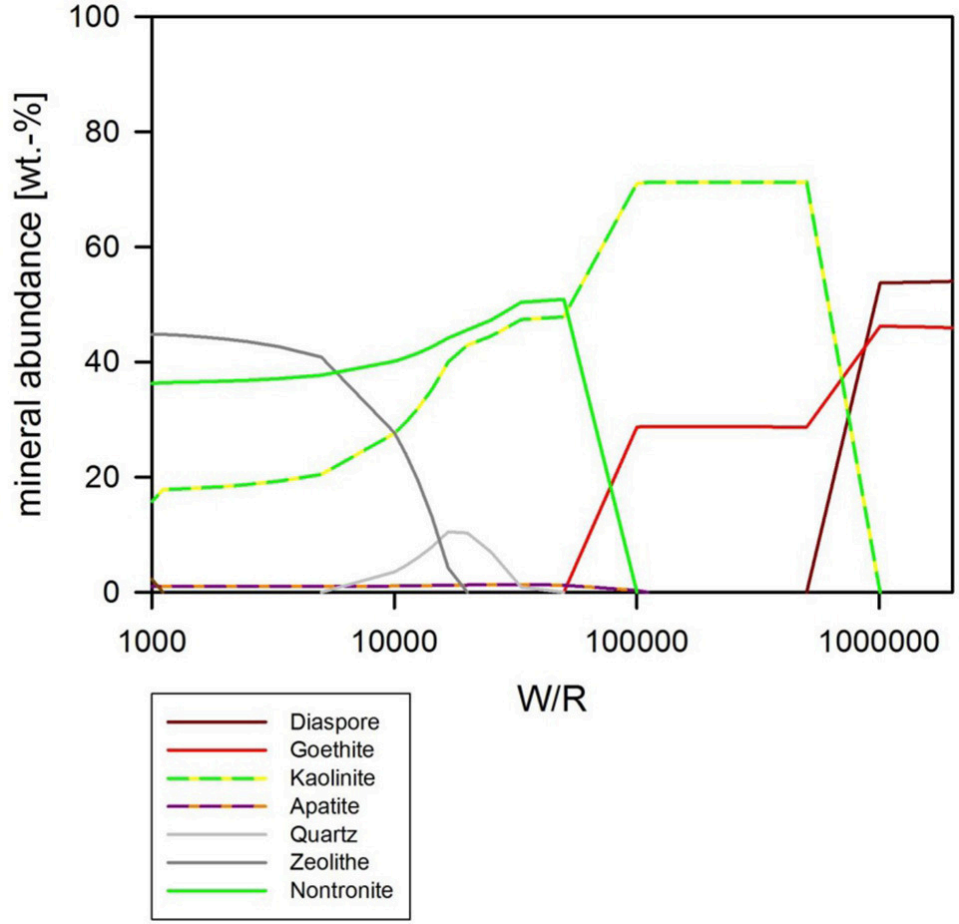
Secondary minerals predicted from the dissolution of analog basalt at 25°C, pH fixed at 7. The mineral precipitates can be divided into three principal assemblages. At low W/R (below about 10,000) zeolites become increasingly important and below about 7,000 are the dominant species. Between about W/R = 10,000 and 900,000, clay minerals (kaolinite and nontronite) are dominating, whereby kaolinite is more prominent at the higher W/R end of that bracket and nontronite at the lower W/R range. At the highest W/R (over 1 Mio) the assemblage contains hydroxides (diaspore and goethite) only.

If pH is held constant at pH 7, the resulting precipitates look somewhat similar to the abiotic model with pH as a free parameter, except chlorite does not form at all, and quartz formation begins at around (W/R)_M_ of 100,000 (Figure 9). In detail, at (W/R)_M_ of >1 Mio, diaspore and goethite precipitate. With increasing rock dissolution Al is no longer precipitated as hydroxide but increasingly consumed by the formation of kaolinite, which forms alongside goethite. In contrast to the previous model, the smectite-group silicate nontronite is added to the assemblage next, together with chlorapatite, while the relative abundance of goethite decreases and eventually becomes unstable. With further increasing dissolution, zeolite (mainly stilbite) formation begins. In summary, many of the same minerals form in both experiments, but the absence of chlorite and the formation of nontronite are important differences between the assemblages that form under the different conditions.

### Biotic simulation—pH buffered at pH 4

**Figure 10:**
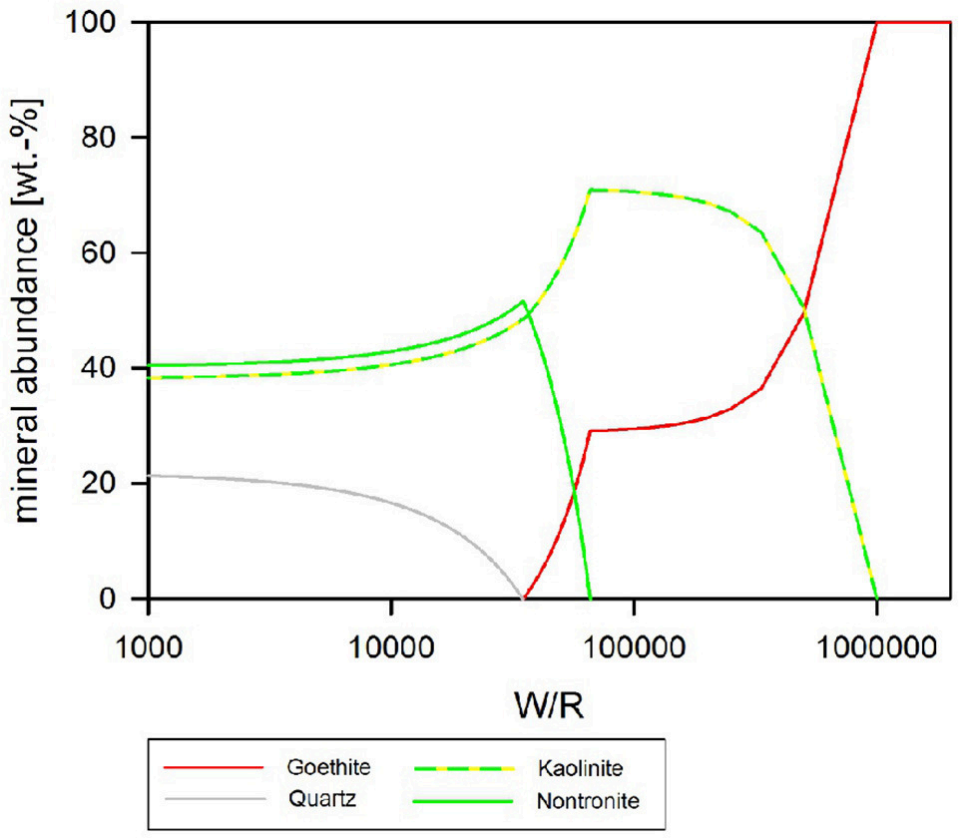
Secondary minerals predicted from the dissolution of analog basalt at 25°C, pH fixed at 4. The precipitated secondary minerals can be grouped in two assemblages: at the highest W/R goethite is the sole precipitate. At lower W/R clays dominate, whereby kaolinte predominantly precipitates at the hither W/R compared to nontronite. Note that quartz starts to precipitate at W/R below about 50,000, but never becomes the dominating species.

If pH is held constant at pH 4, which is the pH during exponential growth, the resulting precipitates have a much simpler composition than in the neutral to alkaline parameter space (Figure 10). In detail, at (W/R)_M_ of >1 Mio, only goethite precipitates while Al stays in solution. Al-precipitation begins with the formation of kaolinite, which forms alongside goethite. In contrast to the previous model, apatite does not form at any (W/R)_M_, but the smectite-group silicate nontronite is added to the assemblage at (W/R)_M_ below 100,000. Again, the relative abundance of goethite sharply decreases and goethite finally becomes unstable at which point quartz forms. Zeolites do not form at low pH.

## Discussion

### Microbially-mediated basalt dissolution

Traditionally, martian analog studies have predominantly focused on chemolithoautotrophic microorganisms from extreme environments (e.g., Fernández-Remolar et al., 2004). However, these lack relevance to the large ancient fluvial systems, which are known to exist on Mars (e.g., Malin and Edgett, 2003; Irwin et al., 2005; Mangold et al., 2012; Williams et al., 2013; Fassett and Head, 2015). Although, chemolithoautotrophic metabolism may have occurred within these fluvial systems, more recent data suggests that chemoorganoheterotrophy was also feasible (Mahaffy et al., 2013; Sutter et al., 2016). Based on the data from Curiosity and our extensive understanding of their role in mineral weathering a chemoorganoheterophic microorganisms (e.g., see review Uroz et al., 2009) was selected for this preliminary study.

Consistent with previous studies, microbial growth enhanced the rate of basalt dissolution in batch culture experiments (e.g., Berthelin and Belgy, 1979; Welch and Ullman, 1993; Wu et al., 2007; Olsson-Francis and Cockell, 2010). The results suggest that, specifically, acidification of the experimental fluid had a positive effect on basalt dissolution, also in agreement with prior studies (e.g., Gislason and Eugster, 1987a,b; Oelkers and Schott, 2001). Acidification is likely to have been caused by the presence of both excess protons and organic acids produced as a by-product of chemoorganoheterophic metabolism (Welch and Ullman, 1993; Vandevivere et al., 1994; Blake and Walter, 1996; Drever and Stillings, 1997). Although, it is difficult to distinguish between organic ligand and proton-mediated dissolution, organic acids have been shown to increase the rate of plagioclase dissolution up to 10 times that achieved in the presence of inorganic acids at the same pH (Welch and Ullman, 1993). At acidic pH, organic acids are more likely to be in the protonated form and thus the proton enhanced pathways of weathering are more favorable, consistent with abiotic inorganic aqueous systems (Buffle, 1990).

*Burkholderia* sp. strain B_33, produced siderophores in the minimal medium, which contained limited iron [the concentration was below the detection limits of the analysis (1 μmol L^−1^)]. However, siderophores were not detected in the medium with the basalt. This is in agreement with previous studies, which were unable to measure siderophore production in the presence of silicate rocks, under laboratory conditions (Frey et al., 2010; Olsson-Francis et al., 2010, 2015). The Fe released from the Fe-bearing minerals (augite, clay) in the rock during dissolution may have inhibited siderophore production. In this study, the basalt contained both augite (8.83% FeO) and vermiculite (20.78% Fe), and ICP-MS data demonstrated that after 1 day the concentration of Fe in the medium was between 3.2 and 5.8 μmol L^−1^. Based on previous work, this is sufficient to inhibit siderophore expression (Olsson-Francis et al., 2010).

### Thermochemical modeling of microbial dissolution

The focus of the thermochemical modeling was the initial stage of the weathering experiments, where supersaturation and the likelihood of secondary phase precipitation was lower; this also reflects the fact that microbial and inorganic weathering in the natural environment often happens in an open system or at least a system open to fluid and the most soluble elements. To compare the experimental and model results, the amount of actual dissolved basalt (W/R)_D_ in the experimental dissolution experiments needed to be deduced. This value was directly comparable to the model water to rock ratio, (W/R)_M_ (Figure 8B). The (W/R)_D_ represented by the vertical lines in Figure 8B was calculated based on the element K, because it is not incorporated into any of the expected mineral precipitates and the intracellular concentration was <2%. Mass balance calculations based on K demonstrate that very little rock dissolution happened over the course of the experiment in either the abiotic [(W/R)_D_ value of ~877,000] or biotic [(W/R)_D_ value of 230,000] experiments. The elemental abundances of the major cations, in the abiotic experiment, were compared with the model at the (W/R)_D_ value of 230,000 [the (W/R)_D_ value determined under biotic conditions].

The abundance of K indicated that dissolution in both the biotic and abiotic experiments was higher than expected from the mass balance calculated from the model. In fact, comparing the measured K abundances with fluid composition of the model indicates that the actual (W/R)_M_ in the biotic experiment would lie in the range of 2,000–2,500. Fe and Ca concentrations predicted by the model are in the range of the measured values, which indicates their precipitation in the minerals and only a small uptake by the bacteria. In contrast, Na, Al, and Si are higher in solution in the experiment than in the model, which indicates inhibition of precipitation. This could occur due to utilization of bio-essential elements required for precipitation, such as oxygen or phosphate, or complexation of the ions by inorganic or organic species produced by the bacterium. The former would reduce the concentration of another component of the precipitating phase, preventing solubility limits to be reached. This shows that, despite the similarities in detected alteration mineral assemblages and the modeled mineral assemblage [compare Figure 7 and Table 5 (experiment) to Figures 8–10 (model)], biological activity changes precipitation characteristics significantly.

The analog rock used here was a naturally occurring basalt, which included clay minerals. This is in accordance with the “mudstones” found by the Curiosity rover at Gale Crater (Vaniman et al., 2014). The observed alteration minerals—detectable through their position on the surface (Figure 7)—agree with the modeled minerals, especially for the low-pH case, where kaolinite forms.

### Bio-signatures and life detection

Field studies have suggested a variety of bio-signatures of past life can be found in basaltic material on Earth. These include morphological fossils in basaltic amygdales and veins (e.g., Schumann et al., 2004; Cavalazzi, 2007), evidence of isotope fractionation in minerals, such as carbonates and sulfides (Demény and Harangi, 1996; Rouxel et al., 2008), or bio-alteration textures, such as potential biogenic tubulars or pitting (Thoreseth et al., 1991; Fisk et al., 1998). Analyses of the Columbia River basalts revealed organic structures, which were interpreted to be bacteria, intermingled with secondary iron oxyhydroxides and ferrous smectites, which suggested that the secondary minerals were caused by microbial activity (McKinley and Stevens, 2000). This study has similarly focused on determining the secondary alteration minerals that could form due to microbially-mediated dissolution of basalt in a Mars-like environment.

A small number of laboratory studies have investigated microbially-mediated basaltic weathering. For example, McKinley and Stevens (2000) studied geochemical modeling in sea water. They showed saturation with respect to Fe(OH)2.7Cl0.3, goethite (α-FeOOH), lepidocrocite (δ-FeOOH) schwertmannite (Fe_8_O_8_(OH)_5_.9(SO_4_)_1.05_), and K-jarosite (KFe_3_(SO_4_)2(OH)_6_ (Daughney et al., 2004). This is broadly consistent with our findings at the highest water to rock ratio (i.e., the formation of kaolinite, and goethite/lepidocrocite), but we note that our system did not contain enough K to form the jarosite modeled by McKinley and Stevens (2000).

Our findings are important for planning and interpreting investigations carried out by rover-based instrumentation currently active on Mars (e.g., CheMin and MastCam and ChemCam passive spectral investigations on Curiosity) and for the selection of future landing sites, e.g., Mawrth Vallis and Oxia Planum currently under discussion for the ESA ExoMars rover (http://exploration.esa.int/mars/53845-landing-site/). Potentially habitable environments may reveal secondary alteration minerals that require the origins (abiotic or biotic) to be unequivocally determined; the combination of thermochemical modeling with simulation studies enables the identification of mineral assemblages that can be used as potential indicators of biotic origin.

Although, the secondary mineral yield was not large enough for detection by XRD in our weeks-to-month long experiments, in a natural martian environment, subsequent generations of microbes would enhance this yield, enabling detection by spacecraft instrumentation.

## Conclusion

In this study, a novel approach was applied combining both laboratory-based experiments and thermochemical modeling to investigate the feasibility of identifying mineralogical bio-signatures that can be used as evidence of life on early Mars. The modeled basalt–fluid reactions resulted in secondary mineral precipitates, which are comparable with the alteration mineralogy found in the rock after the dissolution experiments and the thermochemical models. This confirms the viability of this methodological approach.

The bacterium-enhanced rock dissolution by acidification was likely to be caused by both organic and inorganic acids. The bulk pH of the growth medium may simulate the micro-environment in the natural environment, which is thought to contain concentrated acids and solutes. Comparison of a carefully selected indicator element (K) in the model allowed the deduction of the actual (W/R)_D_. This comparison was used to follow individual element paths to understand how secondary mineral precipitation would differ in biotic and abiotic systems over geological time scales. This approach suggests that biologically-mediated secondary alteration is expected to be “simpler,” consisting of Fe-hydroxide and kaolinite whereas the abiotic system would form chlorite in addition to Fe-hydroxide and kaolinite. This difference is due to differences in chemical diversity and mineralogical complexity; kaolinite has a 1:1 structure containing only Si, Al, and O, whereas chlorite, as a clay mineral, has a 2:1 structure with Si, Al, and O and additional cations (Fe, Mg, Ca). This is important, because most planetary exploration—in terms of spatial coverage—is from orbit using spectral investigations. Those techniques are capable of distinguishing the different groups of clays, but not necessarily subtle variations within a group that might be indicative of the presence or absence of life.

Therefore, due to the different mineral formation pathways utilized in abiotic and biotic systems, deciphering the chemical and mineralogical details necessary for identifying the presence of life is expected to be a task for rover-based investigation. Small sample size, detailed investigations, such as those possible with the rover-based instrumentation currently active on Mars (e.g., CheMin and MastCam and ChemCam passive spectral investigations on MSL), can make use of the fact that, under bacterially-mediated conditions, a less mineralogically and less chemically diverse precipitate is expected. Therefore, this study highlights the differences to look for when investigating a habitable environment for the presence of past life.

Future work will utilize a fresh basalt to minimize the difficulties that the presence of clay minerals had on the model system, e.g., from recent eruptions on Hawaii or Iceland, with polished surfaces singling out grains for pre- and post-experiment mineralogical investigations. The analysis will include instrumentation relevant to past and present life detection missions, including XRD, FTIR, and Raman, to determine whether bio-signatures are detectable. Furthermore, organic molecules will be included in the thermochemical modeling, for example using the program SOLMINEQ88, to investigate whether they inhibit precipitation (Kharaka et al., 1988). This work will further help to specify and quantify the secondary mineralogy that has the potential to serve as inorganic, radiation, and desiccation resistant biosignatures detectable by instruments such as the CheMin Instrument (Blake et al., 2012) on the Mars Exploration rover Curiosity (Blake et al., 2012).

## Author contributions

KOF: Designed the lab experiment, carried out the microbiology analyses, and wrote the manuscript. VP: Carried out the FEG-SEM work. ES: Carried out the geochemical analyses. SS: Ran the models.

### Conflict of interest statement

The authors declare that the research was conducted in the absence of any commercial or financial relationships that could be construed as a potential conflict of interest.
